# Supplementary material for: Deciphering mixed infections by plant RNA virus and reconstructing complete genomes simultaneously present within-host
Source: PLoS One. 2025 Jan 14;20(1):e0311555. doi: 10.1371/journal.pone.0311555 (PMC11731864; doi:10.1371/journal.pone.0311555)
Supplement: S1 Table — The site of initial sample collection IS indicated along with the ID of the field, GPS coordinates and date of sampling. Genbank accession numbers are also mentioned for all the sequences obtained. (DOCX) [file pone.0311555.s001.docx]

**S1 Table.** List of RYMV-infected rice leaves samples from Burkina Faso analyzed in this study.

The site of initial sample collection IS indicated along with the ID of the field, GPS coordinates and date of sampling. Genbank accession numbers are also mentioned for all the sequences obtained

| Code | Site | Field ID | GPS coordinates | Sampling date | Analyzed as | Obtained sequence ID | Genbank Accession |
| --- | --- | --- | --- | --- | --- | --- | --- |
| BF710 | Banzon | na | 11.321 ; -4.805 | 17/10/2016 | ‘greenhouse isolate’ | 2016BF710 | PP489923 |
| BF711 | Banzon | na | 11.322 ; -4.805 | 17/10/2016 |  | 2016BF711 | PP489914 |
| BF706 | Karankasso Sambla | na | 11.247 ; -4.563 | 07/10/2014 |  | 2014BF706 | PP489922 |
| BF707 | Karfiguela | na | 10.683 ; -4.816 | 06/10/2014 |  | 2014BF707 | PP489924 |
| MP1458 | Banzon | BZ11 | 11.335 ; -4.794 | 12/10/2017 | ‘field sample’ | 2017MP1458_h0 | PP996007 |
| MP2967 | Banzon | BZ09 | 11.336; -4.790 | 15/11/2018 |  | 2018MP2967_h0 | PP874882 |
|  |  |  |  |  |  | 2018MP2967_h1 | PP874883 |
| EF0750 | Banzon | BZ21 | 11.323; -4.803 | 08/11/2021 |  | 2021EF0750_h0 | PP874880 |
|  |  |  |  |  |  | 2021EF0750_h1 | PP874881 |
| EF0846 | Banzon | BZ23 | 11.330; -4.809 | 09/11/2021 |  | 2021EF0846_h0 | PP489911 |
|  |  |  |  |  |  | 2021EF0846_h1 | PP489912 |
|  |  |  |  |  |  | 2021EF0846_h2 | PP489915 |
| EF0252 | Badala | BL22 | 11. 368; -4. 373 | 05/10/2021 | ‘greenhouse isolate’ | 2021EF0252 | PP489918 |
| EF0316 | Badala | BL24 | 11.387; -4.363 | 13/10/2021 |  | 2021EF0316 | PP489919 |
| EF0321 | Badala | BL24 | 11.387; -4.363 | 13/10/2021 |  | 2021EF0321 | PP489913 |
| EF0562 | Banzon | BZ04 | 11.328; -4.806 | 08/11/2021 |  | 2021EF0562 | PP489921 |
| EF0580 | Banzon | BZ04 | 11.328; -4.806 | 08/11/2021 |  | 2021EF0580 | PP489916 |
| EF0644 | Banzon | BZ08 | 11.336; -4.802 | 09/11/2021 |  | 2021EF0644 | PP489920 |
| EF0768 | Banzon | BZ21 | 11.323; -4.803 | 08/11/2021 |  | 2021EF0768 | PP489917 |
