# Supplementary material for: Deciphering mixed infections by plant RNA virus and reconstructing complete genomes simultaneously present within-host
Source: PLoS One. 2025 Jan 14;20(1):e0311555. doi: 10.1371/journal.pone.0311555 (PMC11731864; doi:10.1371/journal.pone.0311555)
Supplement: S2 Table — Two recombination events were detected using the RDP4.101 software. The first one was found in only one RYMV haplotype, and the other, already documented in Billard et al 2023 [32], was found in eight viral haplotypes. For each recombination event, we indicate the most likely parents (major and minor), the breakpoints (with their range of confidence in brackets), and the methods detecting the recombination events with the associated P-value. (DOCX) [file pone.0311555.s002.docx]

**S2 Table. Detection of recombination events.** Two recombination events were detected using the RDP4.101 software. The first one was found in only one RYMV haplotype, and the other, already documented in Billard et al 2023 [32], was found in eight viral haplotypes. For each recombination event, we indicate the most likely parents (major and minor), the breakpoints (with their range of confidence in brackets), and the methods detecting the recombination events with the associated *P*-value.

| **Recombination event** | **Recombinants** | **Major parent** | **Minor parent** | **Beginning breakpoint** | **Ending breakpoint** | **Positive methods (*P-*value)** |
| --- | --- | --- | --- | --- | --- | --- |
| 1 | 2018MP2967_h0 | 2017MP1493 | 2021EF0846_h0 | 1 [1-74] | 1014 [929-1095] | R (8.5E-21), G (4.5E-19), B (4.9E-18), M (5.4E-13), C (5.8E-13), S (1.9E-12), 3 (2.7E-32) |
| 2 | 2016BF710, 2016BF711, 2017MP1493, 2017MP1458_h0, 2018MP2967_h0, 2021EF0562, 2021EF0580, 2021EF0750_h0 | 2007Ng105 | 2021EF0750_h1 | 3509 [3424-3555] | - | M (1.2E-5), C (9.1E-3), S (1.7E-27), 3 (1.1E-16) |
