# Supplementary figures and images for: Deciphering mixed infections by plant RNA virus and reconstructing complete genomes simultaneously present within-host
Source: PLoS One. 2025 Jan 14;20(1):e0311555. doi: 10.1371/journal.pone.0311555 (PMC11731864; doi:10.1371/journal.pone.0311555)

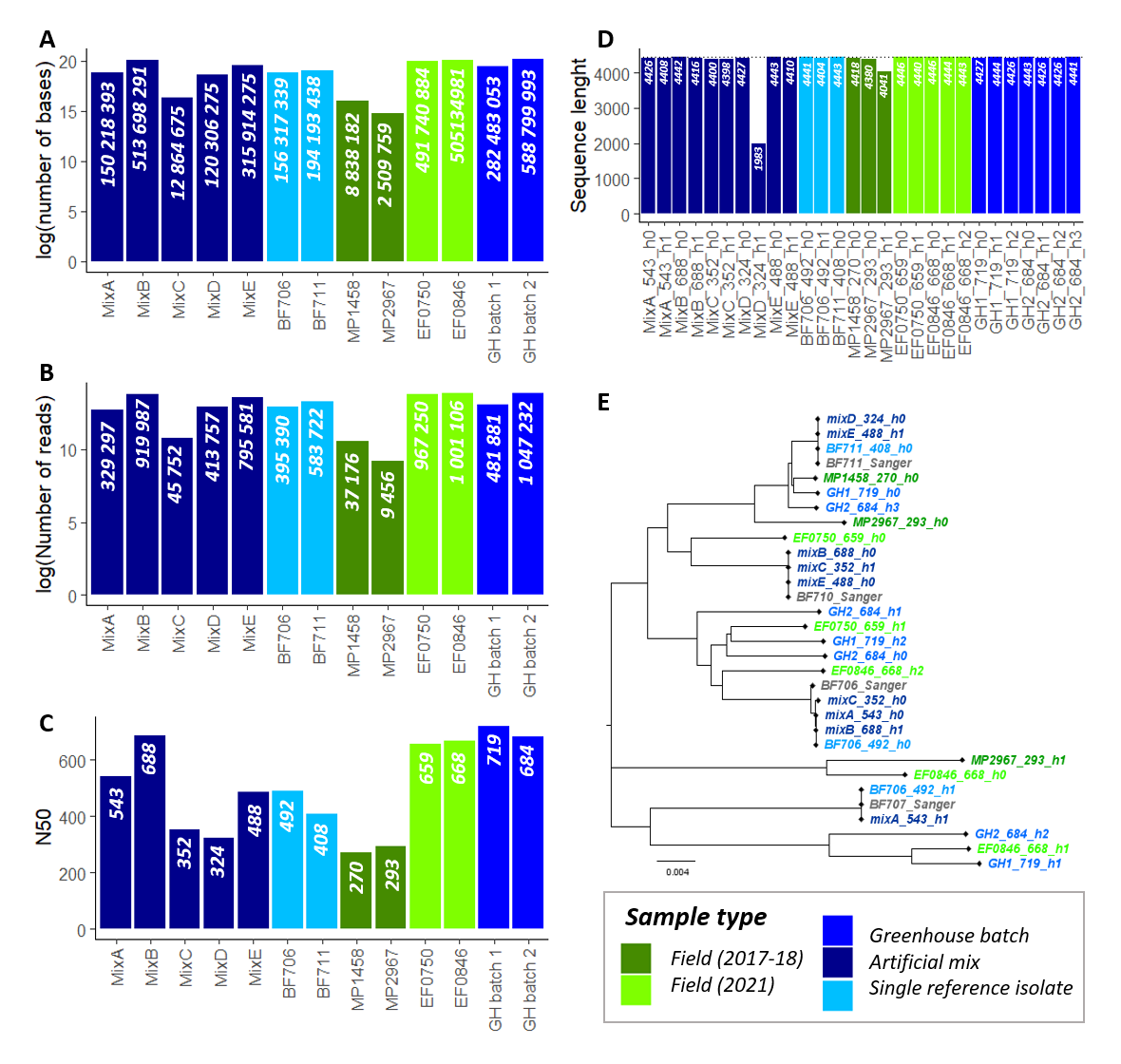

Supplement: S1 Fig — A. Total number of bases obtained for each run (log scale). B. Total number of reads obtained for each run (log scale). C. N50 for each run (defined as the length of the shortest read in the group of longest sequences that together represent half of the nucleotides in the set of sequences). D. Sequence length of the 28 different haplotypes reconstructed by the RVhaplo method over the 13 runs. E. Neighbor-joining phylogenetic tree of the 27 (almost full length) haplotypes obtained, with the four reference genomes obtained by the Sanger technique. The legend, common to the different panels, appears on the bottom right and distinguishes: in green, the field samples, and in blue, the samples corresponding to viral amplification in greenhouses (and then either sequenced either in isolation, or in mixes). (TIF) [file pone.0311555.s003.tif]
