# Supplementary material for: Deciphering mixed infections by plant RNA virus and reconstructing complete genomes simultaneously present within-host
Source: PLoS One. 2025 Jan 14;20(1):e0311555. doi: 10.1371/journal.pone.0311555 (PMC11731864; doi:10.1371/journal.pone.0311555)
Supplement: Attachment — Submitted filename: reviewers_response_vf.docx [file pone.0311555.s004.docx]

PONE-D-24-16411

***Deciphering mixed infections by plant RNA virus and reconstructing complete genomes simultaneously present within-host***

PLOS ONE

Dear Dr. Tollenaere,

Thank you for submitting your manuscript to PLOS ONE. After careful consideration, we feel that it has merit but does not fully meet PLOS ONE’s publication criteria as it currently stands. Therefore, we invite you to submit a revised version of the manuscript that addresses the points raised during the review process.

Please submit your revised manuscript by Jul 06 2024 11:59PM. If you will need more time than this to complete your revisions, please reply to this message or contact the journal office at <a href="mailto:plosone@plos.org">plosone@plos.org. When you're ready to submit your revision, log on to https://www.editorialmanager.com/pone/ and select the 'Submissions Needing Revision' folder to locate your manuscript file.

Please include the following items when submitting your revised manuscript:

- A rebuttal letter that responds to each point raised by the academic editor and reviewer(s). You should upload this letter as a separate file labeled **'Response to Reviewers'**.
- A **marked-up copy of your manuscript** that highlights changes made to the original version. You should upload this as a separate file labeled 'Revised Manuscript with Track Changes'.
- An **unmarked version of your revised paper** without tracked changes. You should upload this as a separate file labeled 'Manuscript'.

If you would like to make changes to your financial disclosure, please include your updated statement in your cover letter. Guidelines for resubmitting your figure files are available below the reviewer comments at the end of this letter.
If applicable, we **recommend that you deposit your laboratory protocols in protocols.io to enhance the reproducibility of your results**. Protocols.io assigns your protocol its own identifier (DOI) so that it can be cited independently in the future. For instructions see: <https://journals.plos.org/plosone/s/submission-guidelines#loc-laboratory-protocols>. Additionally, PLOS ONE offers an option for publishing peer-reviewed Lab Protocol articles, which describe protocols hosted on protocols.io. Read more information on sharing protocols at <https://plos.org/protocols?utm_medium=editorial-email&utm_source=authorletters&utm_campaign=protocols>.
We look forward to receiving your revised manuscript.

Kind regards,

Kandasamy Ulaganathan

Academic Editor

PLOS ONE

**Journal Requirements:**

When submitting your revision, we need you to address these additional requirements.

1. Please ensure that your manuscript meets PLOS ONE's style requirements, including those for file naming. The PLOS ONE style templates can be found at

https://journals.plos.org/plosone/s/file?id=wjVg/PLOSOne**_formatting_sample_main_body.pdf** and 
[https://journals.plos.org/plosone/s/file?id=ba62/PLOSOne_**formatting_sample_title_authors_affiliations**.pdf](https://journals.plos.org/plosone/s/file?id=ba62/PLOSOne_formatting_sample_title_authors_affiliations.pdf)

**This was done**

2. Please expand the acronym “ANR” (as indicated in your financial disclosure) so that it states the name of your funders in full. This information should be included in your cover letter; we will change the online submission form on your behalf.

**This was done**

3. Thank you for stating the following financial disclosure:  "This work was publicly funded through ANR (the French National Research Agency) under the project EVCOPAR (ANR-20-CE35-0004-01)."

Please state what role the funders took in the study. If the funders had no role, please state: "The funders had no role in study design, data collection and analysis, decision to publish, or preparation of the manuscript." If this statement is not correct you must amend it as needed.

Please include this amended Role of Funder statement in your cover letter; we will change the online submission form on your behalf.

**This change was made**

4. Thank you for stating the following in the Acknowledgments Section of your manuscript:

"..This work was publicly funded through ANR (the French National Research Agency) under the project EVCOPAR (ANR-20-CE35-0004-01)." Please note that funding information should not appear in the Acknowledgments section or other areas of your manuscript. We will only publish funding information present in the Funding Statement section of the online submission form. Please remove any funding-related text from the manuscript.

**This was done**

5. When completing the data availability statement of the submission form, you indicated that you will make your data available on acceptance. We strongly recommend all authors decide on a data sharing plan before acceptance, as the process can be lengthy and hold up publication timelines. Please note that, though access restrictions are acceptable now, your entire data will need to be made freely accessible if your manuscript is accepted for publication. This policy applies to all data except where public deposition would breach compliance with the protocol approved by your research ethics board. If you are unable to adhere to our open data policy, please kindly revise your statement to explain your reasoning and we will seek the editor's input on an exemption. Please be assured that, once you have provided your new statement, the assessment of your exemption will not hold up the peer review process.

**All obtained reads are already available in European Nucleotide Archive (**[**https://www.ebi.ac.uk/ena/browser/home**](https://www.ebi.ac.uk/ena/browser/home)**), Bioproject: PRJEB76835, Biosamples: SAMEA115766186 to SAMEA115766198, SRA accessions ERS20300578 to ERS20300591.**

**We also submitted the assembled genomes to Genbank and the accession numbers will be available very soon, with immediate release of the sequences.**

6. We note that you have included the phrase “data not shown” in your manuscript. Unfortunately, this does not meet our data sharing requirements. PLOS does not permit references to inaccessible data. We require that authors provide all relevant data within the paper, Supporting Information files, or in an acceptable, public repository. Please add a citation to support this phrase or upload the data that corresponds with these findings to a stable repository (such as Figshare or Dryad) and provide and URLs, DOIs, or accession numbers that may be used to access these data. Or, if the data are not a core part of the research being presented in your study, we ask that you remove the phrase that refers to these data.

**The mentions to “data not shown” were removed from the manuscript.**

7. Please include your full ethics statement in the ‘Methods’ section of your manuscript file. In your statement, please include the full name of the IRB or ethics committee who approved or waived your study, as well as whether or not you obtained informed written or verbal consent. If consent was waived for your study, please include this information in your statement as well.

**The methods were not submitted to any ethics committee as our research does not directly involve human participants.**

**The rice farmers were asked individually for permission to sample leaves from their fields. The entire project followed the guidelines of the Nagoya protocol regarding access and benefit-sharing.**

**The research on rice health in south-west Burkina Faso (including disease survey and virus sampling) was a collaborative project between the Institut de l'Environnement et des Recherches Agricoles (INERA, Burkina Faso) and the Institut de Recherche pour le Développement (IRD, France). These two institutions formalized their partnership within the International Joint Laboratory Patho-Bios (**[**www.pathobios.com**](http://www.pathobios.com)**) hosted by INERA, mentioned in the acknowledgment section of the manuscript.**

**Such information were added in the revised version, see L134-136:”During field surveys, we asked the farmers individually for permission to sample leaves from their fields. The entire project followed the guidelines of the Nagoya protocol regarding access and benefit-sharing.”**

8. Please upload a new copy of Figure 2 as the detail is not clear. Please follow the link for more information:

https://blogs.plos.org/plos/2019/06/looking-good-tips-for-creating-your-plos-figures-graphics/
https://blogs.plos.org/plos/2019/06/looking-good-tips-for-creating-your-plos-figures-graphics/
[Note: HTML markup is below. Please do not edit.]

**A new version of Figure 2 was now included in the revised version of the manuscript.**

**Reviewers' comments:**

Reviewer's Responses to Questions

**Comments to the Author**

1. Is the manuscript technically sound, and do the data support the conclusions?
The manuscript must describe a technically sound piece of scientific research with data that supports the conclusions. Experiments must have been conducted rigorously, with appropriate controls, replication, and sample sizes. The conclusions must be drawn appropriately based on the data presented.

Reviewer #1: Yes

Reviewer #2: Partly

2. Has the statistical analysis been performed appropriately and rigorously?

Reviewer #1: Yes

Reviewer #2: I Don't Know

3. Have the authors made all data underlying the findings in their manuscript fully available?
The [PLOS Data policy](http://www.plosone.org/static/policies.action#sharing) requires authors to make all data underlying the findings described in their manuscript fully available without restriction, with rare exception (please refer to the Data Availability Statement in the manuscript PDF file). The data should be provided as part of the manuscript or its supporting information, or deposited to a public repository. For example, in addition to summary statistics, the data points behind means, medians and variance measures should be available. If there are restrictions on publicly sharing data—e.g. participant privacy or use of data from a third party—those must be specified.

Reviewer #1: Yes

Reviewer #2: No

4. Is the manuscript presented in an intelligible fashion and written in standard English?
PLOS ONE does not copyedit accepted manuscripts, so the language in submitted articles must be clear, correct, and unambiguous. Any typographical or grammatical errors should be corrected at revision, so please note any specific errors here.

Reviewer #1: Yes

Reviewer #2: Yes

5. Review Comments to the Author

Please use the space provided to explain your answers to the questions above. You may also include additional comments for the author, including concerns about dual publication, research ethics, or publication ethics. (Please upload your review as an attachment if it exceeds 20,000 characters)

**Reviewer #1:**

A pre-print of this manuscript entitled “Deciphering mixed infections by plant RNA virus and reconstructing complete genomes simultaneously present within-host” by these authors available on the https://www.biorxiv.org/content/10.1101/2024.02.29.582683v1.
doi: <https://doi.org/10.1101/2024.02.29.582683>

The manuscript well structured, and the approaches employed for nucleic acid extraction, library preparation, genome detection, bioinformatics and data analysis were deemed suitable.

However, the manuscript requires thorough editing, with specific focus on English grammar, spelling, and sentence structure.

**We used DeepL (**[**https://www.deepl.com/fr/write**](https://www.deepl.com/fr/write)**) to improve the writing of the whole manuscript.**

For example, the caption of Table S2 should be revised.

**The caption of Table S2 was edited, and the revised version is much clear:**

**“S2 Table. Results of recombination dDetection of recombination events. Using RDP4.10 software, Ttwo recombination events were detected using RDP4.101 software., The first one inwas found in only one RYMV genomehaplotype, and the other, already documented in Billard et al 2023 [32], iswas found in eight viral haplotypes. For each of these two recombination events, we are indicated the most likely parents (major and minor depending on the proportion of genomes attributed to each parental genome), the breakpoints, the different methods used to detect recombination, and the associated global P-value are indicated.”**

In the version that I have, the resolution and quality of all graphs and figure are unsatisfactory. The authors should work on making them better.

**The quality of the figures was improved and PACE tool was used to confirm the quality of the revised figures.**

**Reviewer #2:**

Although the idea and the experimental design for this manuscript are interesting, the manuscript in the present form presents important lack of information and some methodological issues.

In the introduction section, the authors never comment transmission mechanisms of this virus that may favour the spreading of mixed infections.

**We agree that the transmission mode of this virus should have been mentioned in the introduction. This is now clearly stated in the revised version, see L81-82: “RYMV is transmitted mechanically, with agricultural practices playing as important role, as well as biotic means including various insect vectors [31].”**

**We do not assume that this transmission mechanisms would specifically favor the spreading of mixed infections (we do not know about this, and think these aspects are poorly documented in the literature in general). However, we argue that the sites presenting high level of genetic diversity are prone to multiple infections in general (see L64), and this is the case for RYMV in Burkina Faso (information added L87-88), but also likely in other area of diversity hotspot such as East Africa.**

In the methods section, methodology used for inoculations is not even mentioned in the text.

**We thank the reviewer for pointing this important missing information. The methodology used for inoculations is now clearly stated in the text, with appropriate reference: see L130-132: “Such infected material derives from experimental infections performed mechanically in controlled conditions [34], symptomatic rice leaves being collected from two to five weeks after inoculation in greenhouses.”**

It is not indicated how many samples were inoculated and what checks have been done to make possible for authors claiming specific strain prevalence in each mixed infection under analysis.

**The number of samples inoculated is not relevant here because we consider as a sample the infected leaves derived from experimental infections.**

**We found that the specific proportion of strains can be derived from ONT sequencing with the proposed methodology because we sequenced mixtures of known proportions, thanks to qPCR estimations (see the section of the material and methods “Sequencing artificial mixed-infection samples”) and the results obtained were in congruence with expectations (see the section “Analyzing artificial mixes results to assess the bioinformatic pipeline” and Table 1).**

It is not clear if they performed any biological and/or technical replicate either for sequencing or for inoculations.

**The inoculation process is very well mastered by the team, it is a quite simple procedure involving mechanical transmission (see doi: 10.21769/BioProtoc.2863), and it is very efficient (close to 100%).**

**For sequencing, we performed technical replicates during the beginning of the process, when evaluating the library preparation procedures (see the section “*Initial sequencing of single isolate to compare three different ONT sequencing strategies*”), as indicated in the text, see L128-129:** “***Each of the three methodologies (VN Primer, RYMV specific primers and direct cDNA) were performed twice*”. For each of the three protocols, the results of the two runs were mostly congruent (see Figure 1).**

**In the next steps of validation of the methodology, we did not perform technical replicates. However, we performed a total of five runs of “artificial mixtures” of isolates. Because the results were satisfactory, we preferred not to perform technical replicates but instead concentrate the efforts to other runs, in order to increase the knowledge on RYMV genomic diversity.**

At L153-154, they speak about sample conservation, commenting a specific batch in comparison to others (?), without explaining why one was better than the other(s).

**We added some specific informations, see L202-204: ”Sample preservation was better for these two samples collected in 2021 (they were kept dried and then frozen at -20 a few weeks after sampling), compared to older (2017-2018) samples (kept dried at ambient temperature for various months or years).”**

In table 1, they claim the use of the two nanopore flowcells (R9 and R10), which have different chemistry leading to different error rates, but this has never been mentioned neither commented in the text. Please, provide an explanation in the text on the reason why you changed chemistry.

**Indeed, we changed nanopore flowcells (R9 for R10) during the course of this study, but this was not in purpose but instead because the kit we used was been discontinued on June 28, 2023 by Oxford Nanopore society, see:**

[**https://nanoporetech.com/products/prepare/discontinued-kits**](https://nanoporetech.com/products/prepare/discontinued-kits)

**We added this information in the text, see L434, “ONT sequencing is a highly dynamic methodology (as exemplified here with the change switching from flow cells R9 to R10 during the course of this study, R9 being no longer available)”.**

The search of recombinants has been done with RDP, but no information has been provided concerning the reference sequence dataset nor the parameters used. It is important to run the RDP analysis using all the full length sequences available, since the results are always biased by the reference dataset.

**RDP analyses were done using the same sequence dataset than phylogeny. This information was added in Method section, see L231: “Finally, potential recombination signals from the complete RYMV genome sequences dataset (i.e. 66 RYMV complete genomes, 19 obtained during this study and the 47 available in public database)”**

As a consequence of the lack of clarity in the methods section, the results appear confused as well, therefore needing a reorganization in line with the information provided in the previous sections.
The discussion needs to be updated in agreement with the modifications of methods and results sections.

**The information that was added in terms of viral multiplication in planta (L132) will help to reader to further understand the manuscript. Also, the modified version of Figure 2 was performed in the aim to make the work performed clearer.**

**Finally, the whole manuscript was carefully revised and we hope some wording will also contribute to improve clarity.**

In addition, L342-343, the authors claim that the methodology they describe "does not capture the viral clouds...", I think that this is not true. Nanopore sequencing is a single molecule sequencing technology, therefore if the authors were able to sequence even just a handful of complete (or almost) genomes, if the error rate is not to high, this could be considered as the viral cloud contained in that specific sample. This check could be particular useful in the case of libraries obtained with R10 chemistry.

**We agree with the reviewer that “Nanopore sequencing is a single molecule sequencing technology”. As a consequence, the different sequencing reads obtained would indeed capture the viral cloud within each specific sample. However, the analysis workflow we used, and in particular RVhaplo, leads to assemble a few genomes from each sample, so that the whole process (wet lab + bioinformatics analysis) results in ignoring the vast majority of variants which are probably a small proportion of the population, and to focus on supposedly independent infections by various RYMV isolates.**

**This was made clearer in the modified version, see L279: “On the other hand, it does not capture the viral clouds of highly related genetic variants generated by within-host replication in high mutation rate RNA genomes is not captured by the proposed methodology (at least at the bioinformatics step, as nanopore single molecule sequencing technology would generate reads of the different variants from the viral cloud). We indeed chose in this study Instead, it allows to focus on supposedly independent infections by various RYMV isolates.”**

L352-353: the claim that nanopore is cost-effective is not totally true. The starter kit could be cheaper that buying an Illumina apparatus, but in terms of library cost, together with the "cheap" nanopore kits, the users have to buy quite a lot of other reagents that, in the end, does not make the sequencing so cheaper compared to an outsourced illumina sequencing. Not to mention the possibility of making mistakes when preparing libraries compared to Illumina standardized systems.

**The cost-effectiveness of our particular methodology is discussed L438.**

**Indeed, the cost of reagents are to be taken into consideration, especially as ribodepletion step is quite costly. However, we carefully compared prices in our conditions and obtained similar pricing rates per genome for nanopore sequencing and Sanger sequencing. We added some details in this respect, see L385: “Including the reagents (especially the ribodepletion step), pricing rate per viral genome of the proposed approach is comparable to” Sanger in our conditions.”**

**Later in the discussion (L441-442) we discuss the opportunity of ONT sequencing for Africa where “Illumina standardized systems” are not deployed. Although it is of course possible to send samples out of the continent for sequencing, we argue that ONT sequencing offers a promising tool for genomics in Africa.**

Finally, the authors did not share any information about the sequences they obtained, I think that if accession numbers are not available they could have at least confidentially shared the sequences identified.

**All obtained reads are already available in European Nucleotide Archive (**[**https://www.ebi.ac.uk/ena/browser/home**](https://www.ebi.ac.uk/ena/browser/home)**), Bioproject: PRJEB76835, Biosamples: SAMEA115766186 to SAMEA115766198, SRA accessions ERS20300578 to ERS20300591. This information was added in the legend of the Table 1 (see L174-176).**

**We also submitted the assembled genomes to Genbank and the accession numbers will be available very soon (and included in Table S1), with immediate release of the sequences.**

**Table 1**. Section "Sequencing information", no data provided for the QC of reads.

For the "RVhaplo results", please provide unit measures.

In the column "distance to reference" please specify the unit measure for this parameter and what is the meaning of the strain within parentheses.

**The legend of Table 1 was carefully re-written.**

**We added to this table a column with the mean quality scores (QC) for each run.**

**Figure 1.** Panel of VNPrimers, homogenize to decimal numeration.

**This was done, see the revised version of Figure 1.**

Figure 2. figure with illegible text

**The figure 2 was completely revised in order to make it clearer.**

**We think these changes make the whole study easier to understand.**

6. PLOS authors have the option to publish the peer review history of their article ([what does this mean?](https://journals.plos.org/plosone/s/editorial-and-peer-review-process#loc-peer-review-history)). If published, this will include your full peer review and any attached files.
If you choose “no”, your identity will remain anonymous but your review may still be made public.
Do you want your identity to be public for this peer review? For information about this choice, including consent withdrawal, please see our [Privacy Policy](https://www.plos.org/privacy-policy).

Reviewer #1: No

Reviewer #2: No

While revising your submission, please upload your figure files to the Preflight Analysis and Conversion Engine (PACE) digital diagnostic tool, <https://pacev2.apexcovantage.com/>. PACE helps ensure that figures meet PLOS requirements. To use PACE, you must first register as a user. Registration is free. Then, login and navigate to the UPLOAD tab, where you will find detailed instructions on how to use the tool. If you encounter any issues or have any questions when using PACE, please email PLOS at <a href="mailto:figures@plos.org">figures@plos.org. Please note that Supporting Information files do not need this step.
