# Supplementary material for: Deciphering mixed infections by plant RNA virus and reconstructing complete genomes simultaneously present within-host
Source: PLoS One. 2025 Jan 14;20(1):e0311555. doi: 10.1371/journal.pone.0311555 (PMC11731864; doi:10.1371/journal.pone.0311555)
Supplement: Attachment — Submitted filename: response_to_reviewers.docx [file pone.0311555.s005.docx]

Dear reviewers,

We thank you for the number of suggestions leading to improve our research article.

This led to a significant improved manuscript, while the conclusions did not change.

All the comments and corrections proposed by the two reviewers were addressed, as described below in blue (line numbers correspond to the marked-up copy of your manuscript that highlights changes made to the original version).

We also added the genbank accession numbers to the S1 Table.

**Reviewer #1:** The authors adequately addressed the reviewers' comments and improved the manuscript accordingly. However, in numerous cases, the lines referenced by the authors in their response to the referee were incorrect, causing difficulties for the reviewing. I think this manuscript may be published in “PLOS ONE” Journal. However, some comments should be addressed before acceptance:

Page 4, Lines 75-78: "The word 'RYMV' was repeatedly used at the beginning of each sentence. Please revise the sentences to avoid starting each one with 'RYMV.'" In addition, remove the comma after "Africa"; replace "in" with "into" for "organized into five overlapping open reading frames"; change "was found to harbor a particularly high RYMV genetic diversity" to "was found to harbor particularly high RYMV genetic diversity"; add "several" before "years".
The paragraph should be revised as follows: Rice yellow mottle virus (RYMV, genus Sobemovirus) causes an endemic rice disease in Africa that poses a threat to the sustainable development of rice production on the continent [30]. The virus is transmitted mechanically, with agricultural practices playing an important role, as well as through biotic means, including various insect vectors [31]. RYMV is a (+) single-strand RNA virus, with a genome of approximately 4450 nt, lacking a 3’ polyA tail and organized into five overlapping open reading frames [30]. A recent study identified a rice yellow mottle disease hotspot in Burkina Faso [32, 33]. This irrigated site was found to harbor particularly high RYMV genetic diversity, with at least four distinct genetic groups coexisting over several years [33].

We agree with the proposition and made this change.

Page 6, Lines 119-125: This paragraph lacks clarity and needs to be rewritten for better understanding. I recommend it be revised as follows:
Each of the three methods (VN primers, RYMV-specific primers and direct cDNA) was performed twice, using RNA extracted from rice plant infected with the BF710 isolate. This infected material came from experimental infections carried out mechanically under controlled conditions [34], using symptomatic rice leaves collected two to five weeks post-inoculation in greenhouses. This approach for viral replication was employed for all samples referred to as “isolates.” The initial inoculum consisted of rice leaves collected from fields in southwestern Burkina Faso. During field surveys, farmers were individually asked for permission to sample leaves from their fields. The entire project adhered to the Nagoya protocol guidelines on access and benefit-sharing.

We agree with the proposition and made this change.

Please remove the vertical lines from the “Table 1”.

This change was done.

-In Table S2: A real recombination event can be detected with high confidence using multiple different methods and with a low associated p-value for each of the methods.
Generally, a recombination event was credibly accepted when detected by three or more methods with p-values < 1.0 × 10–6.
I think the evidence for the second recombination event is not strong or conclusive enough, necessitating further research to confirm its occurrence or understand its significance.
Did the authors consider using other software to verify the recombination event? For example, construct tree using SplitTree software including recombinants.

The second recombination event corresponds to a recombination between two groups (S1wa and S1ca) over the CP-gene coding region (approx. from 3500nt onwards). This recombination event is not newly detected from this manuscript. Indeed, as mentioned L328-330, the second recombination event had been previously described in western Burkina Faso, and reported to as ‘S1bzn’ group (see Billard et al 2023 *Virus Evolution*). A genome sequence was previously obtained by Sanger sequencing, published (sample ID: 2017MP1493, GenBank accession OQ858572.1) and analyzed. In this previous study, a tree was built by excluding the recombinant domain and compared to the CP sequence that corresponds to the recombinant area (see the Supplementary Figure 7 of the same article, Billard et al 2023 *Virus Evolution*) and the recombination profile obtained by RDP analysis was very clear (see the Supplementary Figure 8 of the same article Billard et al 2023 *Virus Evolution*).

The present manuscript included sequences from the literature (47 sequences including the ones described in Billard et al 2023) and new sequences obtained though ONT sequencing (19 haplotypes). When performing the analysis on the new dataset (all 47+19=66 sequences), the above-mentioned recombination event (‘S1bzn’ group) is found, both for the sequence previously published (sample ID 2017MP1493), but also in 7 newly obtained sequences (sample ID: 2016BF710, 2016BF711, 2017MP1458_h0, 2018MP2967_h0, 2021EF0562, 2021EF0580, 2021EF0750_h0).

This recombination event was detected by four methods, three of them (M, S, 3) with very low *P* value (1.2E-5, 1.7E-27 and 1.1E-16, respectively, see the revised Table S2). In addition, the sequences identified as recombinant by RDP analysis (both event 1 and 2) appears as such also in the split network constructed using SplitsTree software (see image below). We are consequently confident in the report of this second recombination event, reported in the results (L328-330) and the discussion (L389-393) sections of the manuscript.

**Reviewer #2:** The corrections made improved the clarity of the manuscript. Please find below some minor suggestions.

L52-53: VANA (Virion-associated nucleic acid)-based
L98: full-length
L168-169: to one known RYMY isolate singly sequenced (runs 6 and 7). Please carefully recheck table caption for English
Table 1, first column header "Run nb", please recheck and correct.
Figure 2 caption: please recheck English and text, particularly the figure title
L429-431: text not clear, please reword
S2 table: n° Recombination event, change to "recombination event". For columns "Beginning breakpoint" and "ending breakpoint", please indicate the meaning of numbers within squared parenthesis

All the suggested changes were done. English wording was checked and improved.
